# Using Genetic Information to Explore Whether Preclinical Alzheimer’s Disease Affects Hearing Difficulty

**DOI:** 10.1093/geroni/igaa057.2922

**Published:** 2020-12-16

**Authors:** Willa Brenowitz, Teresa Filshtein, Kristine Yaffe, Stefan Walter, Thomas Hoffmann, Eric Jorgenson, Rachel Whitmer, Maria Glymour

**Affiliations:** 1 University of California, San Francisco, San Francisco, California, United States; 2 Me, Mountain View, California, United States; 3 UCSF Weill Institute for Neurosciences, San Francisco, California, United States; 4 Rey Juan Carlos University, Madrid, Madrid, Spain; 6 Kaiser Permanente, Oakland, California, United States; 7 University of California Davis, Davis, California, United States

## Abstract

Underlying AD-related neurodegeneration or shared risk factors may influence hearing loss; in an innovative approach we tested whether genetic risk for AD also influences functional hearing loss. We studied 401,084 UK Biobank participants aged 40-70, with Caucasian genetic ancestry, and enrolled 2007-2010. Participants self-reported hearing difficulty and were followed for AD diagnosis until 2018. A genetic risk score for AD (AD-GRS) was calculated as a weighted sum of 23 AD risk variants. In age-, sex-, and genetic ancestry- adjusted models higher AD-GRS was associated with problem hearing in ages 60+(OR= 1.03; 95%CI:1.00, 1.05), but not ages <60 (p>0.05). Using the AD-GRS as an instrumental variable for AD diagnosis, we estimated that incipient AD increased probability of difficulty hearing at enrollment by 45% (95%CI: 1%, 93%). Higher AD-GRS was associated with slightly higher odds of hearing difficulty in older adults. Genetics that predispose for AD also influence late-life hearing difficulty.

